# Tutorial Review for Understanding of Cholangiopathy

**DOI:** 10.1155/2012/547840

**Published:** 2011-08-17

**Authors:** Yasuni Nakanuma

**Affiliations:** Department of Human Pathology, Kanazawa University Graduate School of Medical Science, Kanazawa 920-8640, Japan

## Abstract

The biliary tree consists of intrahepatic and extrahepatic bile ducts and is lined by biliary epithelial cells (or cholangiocytes). There are also peribiliary glands around the intrahepatic large bile ducts and extrahepatic bile ducts. The biliary tree is a conduit of bile secreted by hepatocytes and biliary epithelial cells and also of the peribiliary glands and has several physiological roles. A number of diseases affect mainly the intrahepatic and extrahepatic biliary tree, and, in this special issue, these cholangiopathies are reviewed in detail with respect to genetics, pathogenesis, and pathology. In this paper, the anatomy and physiology of the biliary tree, basic injuries to biliary epithelial cells from stress and bile duct damage, and representative cholangiopathies are briefly reviewed.

## 1. Introduction

A number of diseases affect the biliary tree (cholangiopathies), though the pathological mechanisms involved and the anatomical level of the biliary tree affected vary [1]. For example, small interlobular bile ducts are mainly affected by a Th1-dominated microenvironment and cell-mediated immune response in PBC [2], while a Th2-dominated microenvironment and increased numbers of regulatory T cells are the major features of IgG4-related sclerosing cholangitis which affects mainly the extrahepatic bile ducts [3]. Ischemic damage to the biliary tree is a serious complication in liver transplantations [4]. 

 In this special issue, cholangiopathy with respect to genetics, pathogenesis, and pathology will be discussed in detail. Herein, the anatomy and physiology of the biliary tree, basic injuries to biliary epithelial cells, basic forms of bile duct damage, and etiological classifications of cholangiopathy are reviewed. This tutorial review will be helpful for a better understanding of cholangiopathy. 

## 2. Anatomy and Characteristics of the Biliary Tree

### 2.1. Anatomy

**Figure 1:**
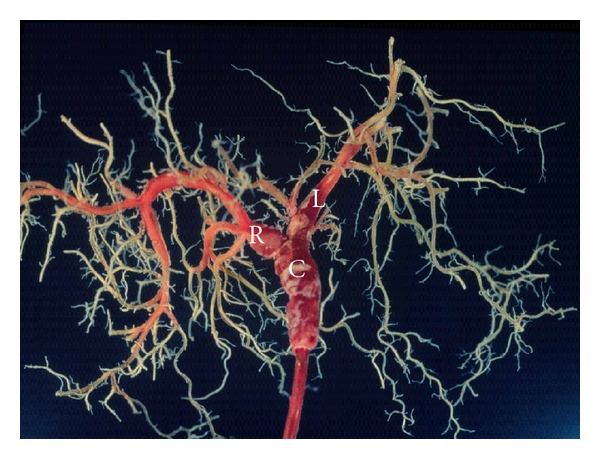
Biliary cast of normal liver. C: common hepatic duct, L: left hepatic duct, and R: right hepatic duct.

**Figure 2:**
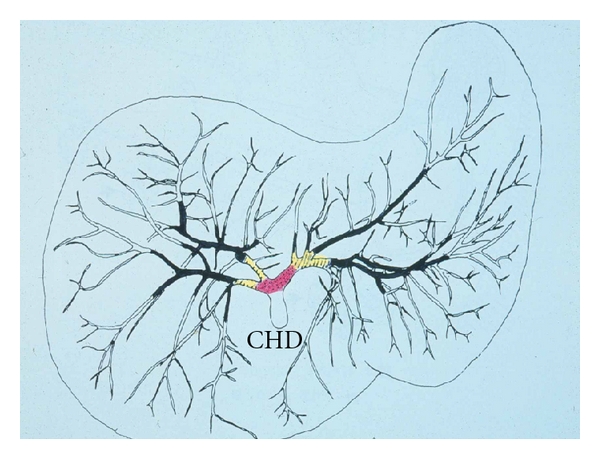
Diagram of the biliary tree. Red: right and left hepatic duct and their confluence, yellow: branches of the right or left hepatic ducts, and black: further branches. CHD: common hepatic duct.

The biliary tree is composed of extrahepatic and intrahepatic bile ducts [5]. The former include the right and left hepatic ducts and their confluence and the common hepatic and bile ducts, while the latter include the bile ducts proximal to the right or left hepatic duct. The intrahepatic branching of the bile ducts is best visualized on a cholangiograph or biliary injection cast (Figures 1 and 2). The extrahepatic bile duct is lined by high columnar epithelial cells, and its wall is composed of dense collagenous tissue harboring scattered smooth muscular elements. 

 The intrahepatic bile ducts can be classified as large and small, though there is no sharp delineation of the various segments [1, 5]. The biliary epithelial cells or cholangiocytes compose approximately 4-5% of liver mass. The large type consists of the right and left hepatic bile ducts and their first to third branches (segmental and area bile ducts). These ducts are grossly visible and belong to the perihilar bile ducts. They are lined by a tall columnar epithelium and surrounded by a dense hypocellular collagenous duct wall. In contrast, small intrahepatic bile ducts, the branches of the large intrahepatic bile duct, are classified into septal and interlobular bile ducts which are visible only under a microscope. While the septal ducts (>100 *μ*m in diameter) are lined by tall columnar cells with basal nuclei, the interlobular bile ducts are lined by cuboidal cells. The fibrous ductal wall is evident in the former like large intrahepatic ducts, but not in the latter. The interlobular bile ducts are connected to the bile canalicular network by ductules (<20 *μ*m diameter) lined by no more than a few minimally differentiated cuboidal cells and the canals of Hering, which are lined partly by biliary epithelium and partly by hepatocytes. The intrahepatic stem cell niches are the canals of Hering in postnatal livers [6]. Bile ductules are very reactive anatomical elements in the liver, and proliferating bile ductules are reportedly involved in the fibrous progression of various chronic liver diseases and are easily identifiable by immunostaining of biliary cytokeratin (CK 7 and 19). 

**Figure 3:**
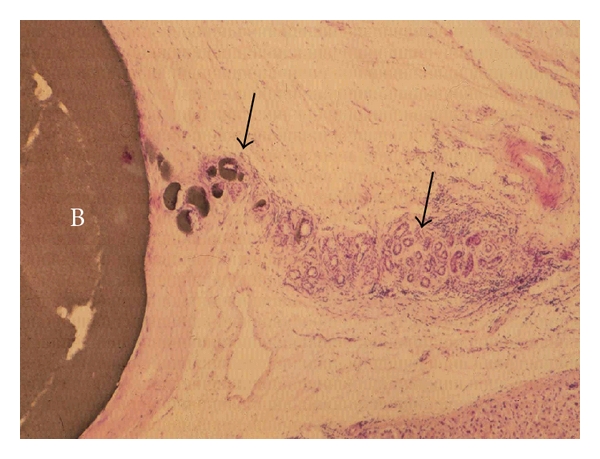
Peribiliary glands. B: bile duct; arrows: peribiliary glands and their conduits.

 Peribiliary glands, the third biliary component, are present within the fibromuscular walls of extrahepatic bile ducts and also along the large intrahepatic bile ducts [1, 5, 7]. Glandular elements are also found at the neck of the gallbladder. Peribiliary glands around the large intrahepatic bile ducts (Figure 3) are subdivided into intramural glands, nonbranching tubular glands, and extramural ramified glands. The latter lie in the periductal connective tissue and, in a three-dimensional model, have a linear distribution along the opposite sides of the bile ducts and indirectly drain into the bile duct lumen via their own conduit. The extramural glands consist of serous and mucous acini. Pancreatic acini without Langerhans' islets are found intermingled with peribiliary glandular acini and are probably an intrinsic component of these glands. The glands are thought to have secretory activities. The extrahepatic stem cell niches are the peribiliary glands deep within the walls of the bile duct [6, 8].

### 2.2. Distribution of Antigens along the Biliary Tree

The individual anatomical components of the biliary tree each have a rather characteristic antigen, probably reflecting a site-specific function [9, 10]. For example, the BECs lining large bile ducts are columnar and mucus is detectable in the supranuclear cytoplasm, but mucin is not detectable in the interlobular bile ducts and bile ductules. In contrast, in the adult liver, the BECs of intrahepatic large bile ducts constantly express MUC3, a membrane-binding type, whereas those of small bile ducts do not. MUC6 is constantly and focally expressed in BECs in the intrahepatic large bile ducts in normal liver. The expression of MUC1, MUC2, and MUC5 was infrequent in normal livers but increased in hepatolithiasis [9]. This study disclosed that the normal biliary tree has a specific expression of blood group antigens at different levels and that this expression is altered under pathologic conditions. In normal livers, large and septal bile ducts expressed A and B antigens in patients with comparable blood groups and also expressed H antigen frequently in patients with blood group O, A, or B and infrequently in patients with type AB. Lea and Leb are expressed in BECs at any level in secretors. As for cytokeratin, CK7 and CK19 are expressed in BECs of the biliary tree and also in peribiliary glands, while EpCAM is expressed in bile ductules. CK8 and 18 are expressed in hepatocytes and also BECs of the biliary tree.

### 2.3. Supply of Blood to the Biliary Tree

The intrahepatic and extrahepatic biliary tract is supplied by a network of fine vessels called the peribiliary vascular plexus (PBP) which exclusively derives from hepatic arterial branches [11–13]. The PVP can be histologically divided into the inner, intermediate, and outer layers, with respect to the bile duct walls [12]. These three layers are well and poorly developed in the large intrahepatic bile ducts and septal bile ducts, respectively, although the PBP around the interloblar bile ducts and bile ductules consists of scattered capillaries with no discernible layers [13]. This plexus has a fern-like appearance around the bile duct under the scanning electron microscope. The PBP drains into the sinusoids through “radicular portal veins” or communicates with portal venous branches through “internal roots” or directly into the hepatic sinusoids in animals and probably in humans.

 The inner layer, a layer of capillaries, is found just beneath the basement membrane of the epithelial layer and is regularly distributed like a chain. Ultrastructurally, the inner capillary layers are composed of fenestrated endothelial cells, and the number of fenestrae with a thin diaphragm is rather high on the capillary side facing the bile duct epithelium [13]. These observations suggest that the PBP, particularly the inner layer, may participate in the physiology of the bile ducts, particularly in the exchange of substances between blood in the peribiliary vascular plexus and bile in the bile ducts and in the supply and drainage of substances to and from the biliary epithelia [12].

### 2.4. Physiological Roles of the Biliary Tree

The biliary tree is lined by specialized epithelial cells called BECs or cholangiocytes [14] and is not only a conduit of bile secreted by hepatocytes and cholangiocytes but also a conduit of the peribiliary glands. The bile ducts and peribiliary glands play a number of physiological roles in the biliary system, contributing to about one-third of total bile secretion, participating in bile acid and water reabsorption, and secretion via transporters, and also mediating immune responses including innate immunity [15]. The primary hepatic bile secreted by hepatocytes is modified by BECs via a series of secretory and absorptive processes that provide additional bile water (BECs secrete ~40% of daily bile production in humans) or secrete HCO_3_^−^ to induce an alkakine state [14]. BECs also interact with the immune system and microorganisms and are also involved in drug metabolism. To accomplish these functions, BECs display morphological and functional heterogeneity along the biliary tree.

### 2.5. Innate Immunity

The biliary tree is essentially sterile under normal conditions, but bile is potentially contaminated by bacterial components such as pathogen-associated molecular patterns (PAMPs) including lipopolysaccharide (LPS) and bacterial DNA originating from intestinal flora, which are actually detectable in bile of patients with chronic inflammatory biliary diseases [16]. In this context, the biliary tract is equipped with defence mechanisms, which are physical (bile flow and biliary mucus), chemical (bile salts), and immunological, such as secretory IgA. BECs also express Toll-like receptors (TLR) and intracellular adaptor molecules and secrete antibiotic peptides and (pro)inflammatory cytokines, thereby participating in the defense of the bile ducts [15]. 

 Nonspecific bactericidal enzymes such as lactoferrin and lysozyme are also detected in the intrahepatic biliary tree, peribiliary glands, and bile [15]. Human *β*-defensins (hBDs) and cathelicidin, another antimicrobial peptide contributing to innate immunity at mucosal surfaces, are expressed in the biliary tree. hBD-1 is constitutively expressed in the biliary epithelium, while hBD-2 is expressed in large intrahepatic bile ducts in extrahepatic biliary obstruction, hepatolithiasis, and, to a lesser degree, PBC and PSC, suggesting a response to local infection or bacterial components, cytokines such as IL-1*β* and TNF-*α*, and/or active inflammation. Cathelicidin is expressed by normal biliary epithelial cells in addition to hepatocytes. Trefoil factor family (TFF) 1, 2, and 3 peptides expressed at the apical surface of the epithelium play a major role in mucosal repair.

 IgA is known to be secreted into bile by binding with the secretory component (SC), and secretory IgA (SIgA) functions in a number of ways to protect the biliary tract. For example, it can directly bind and neutralize bacterial toxins. SIgA can bind to bacteria and prevent their adhesion to the mucosal membrane. Additionally, IgA has been demonstrated to neutralize intracellular microbes and their products. Biliary intraepithelial lymphocytes (bIELs), which are markedly increased in immune-mediated cholangitis, are occasionally encountered in normal intrahepatic bile ducts. Most of them are positive for CD8, some are positive for CD57, and these cells may participate in biliary innate immunity [17].

## 3. Basic Injuries of Biliary Epithelial Cells and Bile Duct Damage

### 3.1. Basic Injuries of BECs

Several pathologic agents and stress affect the intrahepatic and extrahepatic biliary tree including viral, bacterial, and even parasitic infections, oxidative stress, and immunological assaults, as well as biliary epithelial injuries from necrosis, apoptosis, and hyperplasia, and also bile duct damages.

#### 3.1.1. Apoptosis and Necrosis

In some biliary diseases such as primary biliary cirrhosis (PBC) and chronic ductopenic allograft rejection, the ongoing apoptosis of BECs is important for progressive bile duct loss. In H&E stained sections, eosinophilic, shrunken slender cells with pyknotic nuclei in the biliary epithelial layer and fragmented and condensed nuclei in the bile duct lumen can be regarded as apoptotic bodies [2, 15]. Electron microscopically, shrunken BECs with a condensed cytoplasm and pyknotic nuclei are a marker of apoptosis. Apoptosis of BECs can be confirmed using in situ nick-end labelling and immunostaining of single stranded DNA, both of which detect DNA fragmentation. In contrast, the coagulative or lytic necrosis of the biliary epithelium is occasionally encountered in toxic cholangiopathy [18].

#### 3.1.2. Cellular Senescence

Senescent BECs show characteristic features such as an eosinophilic cytoplasm, cellular and nuclear enlargement, multinucleation, and an irregular arrangement with uneven nuclear spacing [19]. Actually, these cells also express cellular senescent markers such as the cell cycle regulators, p16^INK4^ and p21^WAF1/CIP^  , and increased activity of senescence-associated *β*-galactosidase (SA-*β*-gal). Recent studies showed that cellular senescence has at least two pathological effects in the development of biliary diseases: impaired regeneration and senescence-associated secretory phenotypes (SASPs).

**Impaired Regeneration**

Senescent cells no longer have the ability to proliferate and they are irreversibly arrested at the G1 phase of the cell cycle [20]. The expression of senescence-related markers is increased in BECs during early chronic rejection in chronic liver allograft and PBC. Cellular senescence of BECs is involved in impaired regeneration and eventual and progressive bile duct loss in PBC and ductopenic chronic rejection [21, 22]. A relatively insufficient proliferative response of BECs due to cellular senescence (see below) is also responsible for the progressive loss of bile ducts due to apoptosis.

**Senescence-Associated Secretory Phenotypes**

Accumulating evidence suggests that senescent cells remain metabolically active and play an important role in modulating the microenvironment around them by secreting cytokines, chemokines, growth factors, and profibrogenic factors [19]. For example, senescent BECs of PBC expressing CCL2 and CX3CL1 may be involved in the recruitment of monocytes and possibly T lymphocytes into portal tracts, around injured and senescent BECs, and thereby responsible for the development of immune-mediated cholangitis such as PBC [19, 23].

#### 3.1.3. Biliary Epithelial Cell Renewal

The homeostasis of physiological and pathological biliary epithelia operates through a balance between cell loss and cell renewal. Cell loss in the biliary epithelium is mainly due to apoptosis or senescence and mostly regulated by the *bcl-2* family of proteins or senescence-associated factors such as *p16 *and *p21*. The biliary epithelial cells of bile ductules or small bile ducts may be replenished by bile ductular cells or hepatic progenitor cells in the canal of Hering, though such processes may be unlikely in the intrahepatic large bile ducts. As mentioned, the peribiliary glands themselves or progenitor cells located in these glands may be involved in renewal of the biliary epithelium of intrahepatic large bile ducts and extrahepatic bile ducts and also proliferation of the epithelia lining these bile ducts [7, 8].

#### 3.1.4. Biliary Epithelial Hyperplasia

Inhibition of the apoptotic or senescent process in the biliary epithelia may cause hyperplasia with an increased risk of neoplastic transformation [24]. Hyperplasia of lining epithelia of the septal and large bile ducts manifests as micropapillary projections or as a stratification of the epithelium with or without dilatation of the duct lumen. Peribiliary glands, intramural or extramural, also show hyperplasia and proliferation and participate in the secretion of neutral, carboxylated, and sulphated mucins into the bile duct lumen. When prominent, in particular with *Clonorchis sinensis* infections or hepatolithiasis, the term adenomatous hyperplasia or chronic proliferative cholangitis has been used. As for the proliferation and hyperplasia of bile ductules and small interlobular bile ducts, they appear tourtous and increase in their number in the portal tracts. Some of these lesions are included in the so-called ductular reactions [25].

#### 3.1.5. Metaplasia

Several kinds of metaplasia are reported in the biliary epithelium of the intra- and extrahepatic biliary tree, usually in cases of chronic biliary diseases such as hepatolithiasis, parasitic cholangitis, and primary sclerosing cholangitis (PSC). *Gastrointestinal metaplasia* resembling pyloric glands and goblet cells is not infrequently seen in chronically inflamed large bile ducts and peribiliary glands. This change is associated with the aberrant expression of gastric type mucus core protein (MUC) 5AC and MUC6 and also intestinal type MUC2. Such gastric and intestinal mucin is involved in lithogenesis in hepatolithiasis [9]. The so-called intramural glands with a gastric pyloric gland-like appearance are increased in long-standing biliary diseases and may reflect invagination of the biliary epithelium with gastrointestinal metaplasia. *Goblet cells* are occasionally encountered among bile duct-lining cells and also in peribiliary glands. *Intestinal metaplasia *with Paneth cells may also be encountered in the peribiliary glands. The expression of other molecules in intrahepatic large bile ducts, such as REG I and trefoil factors, appears to be related to intestinal or gastric metaplasia. While *pancreatic acinar metaplasia *is also reported infrequently in PSC, its differentiation from heterotopic pancreatic acini is controversial. *Hepatocytic metaplasia* occurs in interlobular bile ducts and bile ductules in various pathological situations but remains of unknown significance. *Squamous metaplasia* is rarely encountered in long-standing inflammation of large bile ducts such as PSC or in the lining of biliary cysts.

#### 3.1.6. Biliary Intraepithelial Neoplasm (BilIN)

Chronic biliary diseases such as hepatolithiasis and PSC are occasionally complicated by cholangiocarcinoma. In such cases, dysplastic or early neoplastic lesions are known to precede the invasive cholangiocarcinoma. Such biliary epithelial lesions are known as dysplasia or atypical hyperplasia of the biliary epithelium and characterized by atypical, enlarged, and hyperchromatic nuclei, an increased nucleocytoplasmic ratio, and a loss of polarity [5, 26]. Usually either micropapillary or flat lesions affect a portion or the circumference of the bile duct. These lesions were proposed to be called biliary intraepithelial neoplasm (BilIN), and this terminology was recently adopted by WHO [26]. They are divided into three grades according to cellular and structural atypia; BilIN-1, BilIN -2, and BilIN -3. In BilIn-1, cellular/nuclear atypia are mild or moderate but not enough for overt malignancy, and cellular polarity is minimally disturbed and corresponding to low-grade dysplasia. In BilIN-2, cellular/nuclear atypia are evident but not marked enough for overt malignancy, and the disturbance of cellular polarity is mild or focal, corresponding to high-grade dysplasia. BlIN-3 shows cellular/nuclear atypia corresponding to overt malignancy, and cellular polarity is diffusely disturbed, corresponding to a so-called carcinoma in situ of the biliary tract [26]. BilIN-1, BilIN -2, and BilIN -3 are seen in both large intrahepatic and extrahepatic bile ducts, peribiliary glands, and gallbladder and considered to reflect a multistep neoplastic transformation of the biliary epithelium.

### 3.2. Basic Pathology of Bile Duct Damage

In the biliary tree, there are several types of bile duct damage such as cholangiopathies and cholangitis. Representative pathological features of the biliary tree are as follows.

#### 3.2.1. Cholangitis and Its Classification

Cholangitis is characterized by biliary epithelial damage with inflammatory cell infiltration. Some cholangitis is also associated with ductal and periductal fibrosis. It occurs along the biliary tree, and the term cholangitis is used for inflammatory damage to bile ductules. 

 Cholangitis can be histologically classified into suppurative and nonsuppurative forms. *Suppurative cholangitis* implies the presence of numerous polymorphonuclear cells around and within the wall as well as within the lumen of the ducts. This may involve ducts of any size and is occasionally associated with abscess formation—cholangitic abscess. A microbial infection is often responsible, but the change also occurs in the presence of sterile bile, particularly after bile extravasation. The release of chemokines or cytokines is the likely cause in some cases. 

 “*Nonsuppurative cholangitis*” includes a spectrum of bile duct inflammation which may be granulomatous cholangitis, lymphoid cholangitis, fibrous cholangitis, and pleomorphic cholangitis according to the predominant type of inflammatory reaction present [27]. *Granulomatous cholangitis *almost always seems to be destructive. This type involving the interlobular bile ducts constitutes the hallmark of PBC and is also found in drug-induced liver disease and sarcoidosis. The other types can be either destructive or nondestructive. *Lymphoid cholangitis* refers to a close association between duct branches, usually interlobular bile ducts, and lymphocytic aggregates, which may show a follicular arrangement. This is found in PBC and PSC with concomitant bile duct destruction or in nonbiliary disorders, in particular autoimmune and viral hepatitis C. *Pleomorphic cholangitis* is associated with inflammatory cell infiltration. All other types of cholangitis are found in CAH, PBC, PSC, and other liver diseases. *Fibrous cholangitis (also called sclerosing cholangitis) *with evident ductal fibrosis develops as a consequence of long-standing bile duct inflammatory, obstruction, or ischemic injury; it can be obliterative or nonobliterative. BECs show variable damage. The former is characteristic of PSC, though, in our experience, it may be seen in acquired forms of sclerosing cholangitis too. BECs of obliterative type are actually lost in fibrous lesions, appearing as a fibrous core. Sclerosing cholangitis with bile duct obliteration suggests a diagnosis of PSC in adults.

#### 3.2.2. Bile Duct Sclerosis

In long-standing sclerosing cholangitis and also in other biliary diseases such as ischemic cholangitis, the bile duct wall shows a marked deposition of collagen fiber (bile duct sclerosis). The affected bile ducts in sclerosing cholangitis show a marked increase in the number of c-kit receptor-expressing mast cells which secrete fibrogenic factors such as histamine, basic fibroblast growth factor (bFGF), and/or tumour necrosis factor-alpha (TNF-*α*). The biliary epithelium itself produces and secretes fibrogenic substances such as bFGF, transforming growth factor-beta (TGF-*β*), and platelet-derived growth factor (PDGF), as well as basement membrane proteins and extracellular matrix proteins. In biliary atresia, BECs of the affected bile ducts variably express mesenchymal markers such as vimentin and might have acquired phenotypes of mesenchymal cells, though distinct morphological epithelial mesenchymal transition (EMT) of biliary epithelium is hardly recognizable [28, 29]. In all forms of bile duct sclerosis, a marked attenuation of the peribiliary vascular plexus is seen within the sclerotic duct wall, but it remains unknown whether these changes are secondary to, or responsible for, the bile duct fibrosis.

#### 3.2.3. Bile Duct Loss or Ductopenia

The balance of cell death or dropout due to apoptosis or necrosis and the regeneration of lining biliary epithelia is important for the maintenance of bile ducts, and apoptotic activity that exceeds the proliferative response of bile duct cells results in progressive ductopenia. Ductopenia is defined as a loss of bile ducts from the portal tract in which hepatic arterial branches and bile ducts of similar size run parallel. Thus, portal tracts without evident bile ducts indicate a loss of bile ducts. Immunostaining of biliary cytokeratins such as CK7 and CK19 is helpful for the recognition of bile ducts. Ductopenia is usually defined as the absence of interlobular bile ducts in at least 50% of portal tracts. Extensive ductopenia is usually associated with chronic cholestasis and biliary fibrosis. Ductopenia is typically found during chronic liver allograft rejection with chronic cholestasis and also the advanced stages of PBC.

#### 3.2.4. Mucobilia and Hemobilia

Mucin is impacted in the duct lumen and this is occasionally marked, leading to leakage and extravasation with the formation of mucus lakes. Drainage of mucin from Papilla of Vater is also a clinical manifestation of mucobilia, as seen in intraductal papillary mucinous neoplasms of the pancreas. Mucobilia is usually found in the neoplastic bile ducts and nonneoplastic bile ducts of “intraductal papillary neoplasms of the bile duct” (formerly known as “biliary papillomatosis”) or mucin-producing bile duct tumors [26, 30]. When such changes are encountered in nonneoplastic biliary diseases such as PSC and hepatolithiasis, usually microscopic neoplastic biliary lesions are found in the affected bile ducts.

 In cases of hemobilia, impacted erythrocytes are encountered in bile duct lumens. Recent endoscopic or surgical biliary manipulations in association with a primary or secondary malignancy may be underlining diseases for hemobilia.

#### 3.2.5. Ductular Reaction

To date, many pathological terms such as oval cell proliferation, intermediate cells, and atypical bile ductular proliferation have been used to describe the “increased ductule-like cells or clusters of small epithelial cells different from mature hepatocytes” in the portal tract or the periportal area. This is a reaction of the ductular phenotype, possibly but not necessarily of ductular origin, commonly seen in many kinds of acute and chronic hepatobiliary diseases. Recently, an international working group proposed the term “ductular reaction” for this lesion [25]. “Ductular reaction” implies a reaction of ductular phenotype, possibly but not necessarily of ductular origin. The epithelial component of a ductular reaction may actually derive from several sources: not only from the proximal branches of the biliary tree but also from the circulation (often if not always from bone marrow) and from biliary metaplasia of hepatocytes. “Reaction” encompasses the complex of stroma, inflammatory cells, and other structures of diverse systems, all of which participate in the reactive lesion. Bile ductular reaction is usually characterized by increased numbers in the periportal and portal areas and a common and frequent process in a number of hepatobiliary diseases. 

 A ductular reaction itself is heterogeneous in its development and has many meanings. There are several reports that bile ductules are very reactive anatomical elements in the liver, and proliferated bile ductules are involved in the progression of various chronic liver diseases. Our recent studies showed that bile ductular cells in PBC, PSC, and also NAFLD may undergo cellular senescence, and these cells could produce and secrete biologically active molecules and thereby be involved in hepatic fibrogenesis and other pathologic features of the liver. 

#### 3.2.6. Ductal Plate Malformation

Ductal plate malformations (DPMs), which are different from reactive changes of bile ducts or ductules, develop as a result of a remodeling failure of the ductal plate followed by the development of intrahepatic bile ducts. DPMs are characterized by increased numbers of abnormal bile duct-like structures and show a bridge-like structure in the dilated lumen and bulbar protrusion of biliary epithelia [31]. DPMs are observed in congenital hepatic fibrosis and Caroli's disease, biliary atresia, and other fibropolycystic liver diseases.

## 4. Cholangiopathies

Diseases that mainly target the biliary tree (cholangiopathies) can be divided into several categories according to the pathogenetic mechanism involved (Table 1). However, in many cholangiopathies, more than one pathogenetic mechanism is operative. 

Etiologic classification of cholangiopathy.

### 4.1. Immune-Mediated Cholangiopathies

The biliary tree could be affected by immunological assaults, and lymphoplasmacytic infiltration is evident around the damaged bile ducts. Primary biliary cirrhosis (PBC) and primary sclerosing cholangitis (PSC) are representative immune-mediated cholangiopathies [32]. Autoimmune pathogenesis is operative in PBC and PSC. There is a mixture of immunocompetent cells in the affected bile ducts, and CD3+, CD4+, and CD8+ T cells that bear the T-cell receptor *α*/*β* are predominant in PBC, supporting that Th1 immune response-predominant cytotoxicity and/or cytokine release are involved in the pathogenesis of the bile duct lesions of PBC. HLA-class II antigens are aberrantly expressed in the affected bile ducts of PBC, PSC, and chronic allograft rejection [33]. Biliary innate immunity is also involved in the pathogenesis of cholangiopathies in patients with PBC and biliary atresia (BA) [9]. BECs possess an innate immune system consisting of the Toll-like receptor (TLR) family and recognize pathogen-associated molecular patterns (PAMPs). In PBC, CD4-positive Th17 cells characterized by the secretion of IL-17 are implicated in the chronic inflammation of bile ducts, and the presence of Th17 cells around bile ducts is causally associated with the biliary innate immune responses to PAMPs. In BA characterized by a progressive, inflammatory, and sclerosing cholangiopathy, dsRNA viruses are speculated to be an etiological agent and to directly induce enhanced biliary apoptosis via the expression of tumor necrosis factor-related apoptosis-inducing ligand (TRAIL). Moreover, the epithelial-mesenchymal transition (EMT) of biliary epithelial cells is also evoked by the biliary innate immune response to dsRNA. In addition, intrahepatic small bile ducts and bile ductules are a main target in graft-versus-host disease and also hepatic allograft rejection. Recent studies showed that IgG4-related sclerosing cholangitis is also associated with altered immunity. Upregulation of regulatory T cells (Tregs) associated with Th2 predominance is reportedly important in the pathogenesis of IgG4-related sclerosing cholangitis [3]. The anatomical level of the biliary tree affected is different among these immune-mediated cholangiopathies. Interestingly, the peribiliary glands are also involved in PSC, graft-versus-host disease, and IgG4-related sclerosing cholangitis.

### 4.2. Infectious Cholangiopathies

The biliary tree is affected by several types of infectious diseases, such as bacterial, fungal, protozoan, parasitic, and viral cholangitis. Stagnation of bile due to biliary stenosis or obliteration is followed by bacterial cholangitis, frequently with sepsis or abscess formation. Parasitic infections are also reported in the biliary tract including the liver, and liver flukes such as *Clonorchis sinensis* and *Opisthorchis viverrini* are endemic in East Asia, particularly northern Thailand and some parts of Korea, and cholangiocarcinoma is a serious complication of parasitic cholangitis [26]. Hepatolithiasis is predominantly a disease of the Far East and is causally also related to infectious cholangitis, especially bacterial cholangitis [27, 34]. Mucin plays an important role in the development of hepatoliths, which are formed within the intrahepatic large bile ducts. Clinically, patients may present acutely with recurrent bacterial cholangitis and its possible complications, such as liver abscesses and septicemic shock, or with chronic complications, such as cholangiocarcinomas and intraductal papillary neoplasms [35]. Pathologically, it is characterized by pigmented calcium bilirubinate stones within dilated intrahepatic bile ducts featuring chronic inflammation, mural fibrosis, and proliferation of peribiliary glands, without extrahepatic biliary obstruction. A transient viral infection such as type A rhesus rotavirus and type 3 reovirus is reported as an initiating mechanism of biliary atresia (BA), particularly perinatal type.

### 4.3. Genetic Cholangiopathies

Genetic alterations affecting the biliary tree manifest as biliary dilatation, bile duct paucity, obstruction, proliferation, stone formation, and so on. Caroli's disease with congenital hepatic fibrosis (CHF) is a representative genetic cholangiopathy. Some cases of biliary atresia (BA) also belong to this category. The former is characterized by multiple saccular dilatations of the intrahepatic bile ducts. Caroli's disease with CHF belongs to autosomal recessive polycystic kidney disease (ARPKD) with ductal plate malformation characterized by a disordered remodeling of the intrahepatic biliary tree [28]. Disordered cell kinetics, including the apoptosis of biliary epithelial cells (BECs), may be significantly related to ductal plate malformation, and laminin and type IV collagen levels were reduced in the basement membrane of intrahepatic bile ducts of ARPKD; such a reduction is an additional factor for the dilatation of bile ducts. Paucity of the intrahepatic bile ducts is a genetic cholangiopathy [36]. For example, Alagille syndrome with a mutation in a ligand for the Notch protein is characterized by paucity of intrahepatic bile ducts and other anomalies. Cystic fibrosis (CF) due to a mutation in the cystic fibrosis transmembrane conductance regulator (CFTR) is associated with focal biliary fibrosis, and the bile duct and ductules are filled with pink and amorphous secretions. Low phospholipid-associated cholelithiasis (LPAC) is characterized by a low biliary phospholipid concentration with symptomatic and recurring cholelithiasis, and LPAC syndrome is associated with mutations of the adenosine triphosphate-binding cassette, subfamily B, member 4 (ABCB4) gene encoding the hepatobiliary phospholipid translocator multidrug resistance protein 3 [37]. This causes recurrent cholelithiasis, continuous irritations of the biliary tract with cholangitis, chronic cholestasis, and even biliary cirrhosis.

### 4.4. Ischemic Cholangiopathies

Ischemic cholangiopathy is defined as focal or extensive damage to bile ducts due to an impaired blood supply [11]. Most causes of bile duct ischemia are iatrogenic, though some systemic vascular diseases also cause this type of cholangiopathy. This entity may be observed in various circumstances and is of clinical importance for practitioners involved in gastroenterology, oncology, abdominal surgery, and liver transplantation. Ischemic bile duct injury may occur when small hepatic arteries or the peribiliary vascular plexus are injured or when all possible sources of arterial blood supply are interrupted. Ischemic biliary injury may take the form of bile duct necrosis, bile leakage, biloma, bile duct fibrosis, or stenosis. Bile duct necrosis and bilomas develop predominantly where there is an abrupt and complete interruption of the arterial blood supply, for example, when HA thrombose in a liver transplant recipient. On the contrary, fibrous stenoses develop where there is progressive injury to the hepatic arterioles, for example, after several courses of intra-arterial chemotherapy. Cholangiographic findings include diffuse and multiple bile ducts lesions. Ischemic cholangiopathy is a serious complication during liver transplantation [4]. When biliary drainage or reconstruction is not possible or has failed, liver transplantation is the only potential cure.

### 4.5. Drug- or Toxin-Induced Cholangiopathies

Bile ducts, particularly interlobular bile ducts, are occasionally affected by drug-induced hepatobiliary damage, various bile duct injuries, various types of cholangitis, and bile duct loss (drug-induced cholangiopathy) [38]. This type of cholangitis is not infrequently associated with cholestasis. Some cases presenting with progressive ductopenia and cholangitis and prolonged cholestasis mimic PBC and also PSC. While the mechanism of drug-induced cholangitis remains speculative, immune-mediated processes including hypersensitivity may be operative. Some forms of drug-induced cholangiopathy develop after hepatic arterial infusion of floxuridine (FUDR) (floxuridine- (FUDR-) induced cholangiopathy). Ischemic changes to the peribiliary vascular plexus may be at least partly involved in this type of cholangiopathy. Although BECs have low metabolic activity compared with hepatocytes, cytotoxic or cytopathic bile duct injury has been produced experimentally or accidentally by toxic substances such as *α*-naphthylisothiocyanate, 4,4′-diaminodiphenylmethane, and paraquat (toxin-induced cholangiopathy) [18].

 In conclusion, the anatomy and physiology of the biliary tree, basic injuries to biliary epithelial cells, basic forms of bile duct damage, and etiological classification of cholangiopathy were reviewed. This tutorial review will be helpful for better understanding cholangiopathies.

## Figures and Tables

**Table 1:** 

(1) Immune-mediated cholangiopathy
(2) Infectious cholangiopathy
(3) Genetic cholangiopathy
(4) Ischemic cholangiopathy
(5) Drug- or toxin-induced cholangiopathy
